# Mutational Dissection of Telomeric DNA Binding Requirements of G4 Resolvase 1 Shows that G4-Structure and Certain 3’-Tail Sequences Are Sufficient for Tight and Complete Binding

**DOI:** 10.1371/journal.pone.0132668

**Published:** 2015-07-14

**Authors:** Philip J. Smaldino, Eric D. Routh, Jung H. Kim, Banabihari Giri, Steven D. Creacy, Roy R. Hantgan, Steven A. Akman, James P. Vaughn

**Affiliations:** 1 Department of Cancer Biology, Wake Forest School of Medicine, Winston-Salem, North Carolina, 27157, United States of America; 2 Department of Chemistry, Furman University, Greenville, South Carolina, 29613, United States of America; 3 Harmonyx Diagnostics, Cordova, Tennessee, 38016, United States of America; 4 Department of Biochemistry, Wake Forest School of Medicine, Winston-Salem, North Carolina, 27157, United States of America; 5 Department of Hematology and Oncology, Roper St. Francis Hospital, Charleston, South Carolina, 29401, United States of America; Virginia Commonwealth University, UNITED STATES

## Abstract

Ends of human chromosomes consist of the six nucleotide repeat d[pTTAGGG]_n_ known as telomeric DNA, which protects chromosomes. We have previously shown that the DHX36 gene product, G4 Resolvase 1 (G4R1), binds parallel G-quadruplex (G4) DNA with an unusually tight apparent K_d_. Recent work associates G4R1 with the telomerase holoenzyme, which may allow it to access telomeric G4-DNA. Here we show that G4R1 can tightly bind telomeric G4-DNA, and in the context of the telomeric sequence, we determine length, sequence, and structural requirements sufficient for tight G4R1 telomeric binding. Specifically, G4R1 binds telomeric DNA in the K^+^-induced “3+1” G4-topology with an apparent K_d_ = 10 ±1.9 pM, a value similar as previously found for binding to unimolecular parallel G4-DNA. G4R1 binds to the Na^+^-induced “2+2” basket G4-structure formed by the same DNA sequence with an apparent K_d_ = 71 ± 2.2 pM. While the minimal G4-structure is not sufficient for G4R1 binding, a 5’ G4-structure with a 3’ unstructured tail containing a guanine flanked by adenine(s) is sufficient for maximal binding. Mutations directed to disrupt G4-structure similarly disrupt G4R1 binding; secondary mutations that restore G4-structure also restore G4R1 binding. We present a model showing that a replication fork disrupting a T-loop could create a 5’ quadruplex with an opened 3’tail structure that is recognized by G4R1.

## Introduction

Telomeres are specialized nucleic acid/protein structures that “cap” the ends of chromosomes, protecting them from chromosomal end-joining, recombination, and degradation [1,2]. Human telomeric DNA consists of 1–15 kilobases of double-stranded tracts of d[pTTAGGG]_n_ repeats that terminate in a ca. 50–200 nt single-stranded G-rich 3’overhang at the end of each chromosome [3,4]. The 3’ termini of telomeric DNA cannot be replicated completely by conventional DNA polymerases, resulting in progressively shorter telomeres with each round of replication. Therefore, somatic cells can undergo only a limited number of divisions before the telomeres become critically short, causing them to lose their protective qualities and resulting in senescence or apoptosis signaling within the cell [2,5]. The ribonucleoprotein (RNP) reverse transcriptase known as telomerase is primarily responsible for preventing this loss and for maintaining telomere length. Telomerase is overexpressed in greater than 85% of all cancers and undetectable in most adult tissue [6], making telomerase and telomere biology a topic of intense focus for the development of targeted cancer therapies [4,7,8].

**Fig 1:**
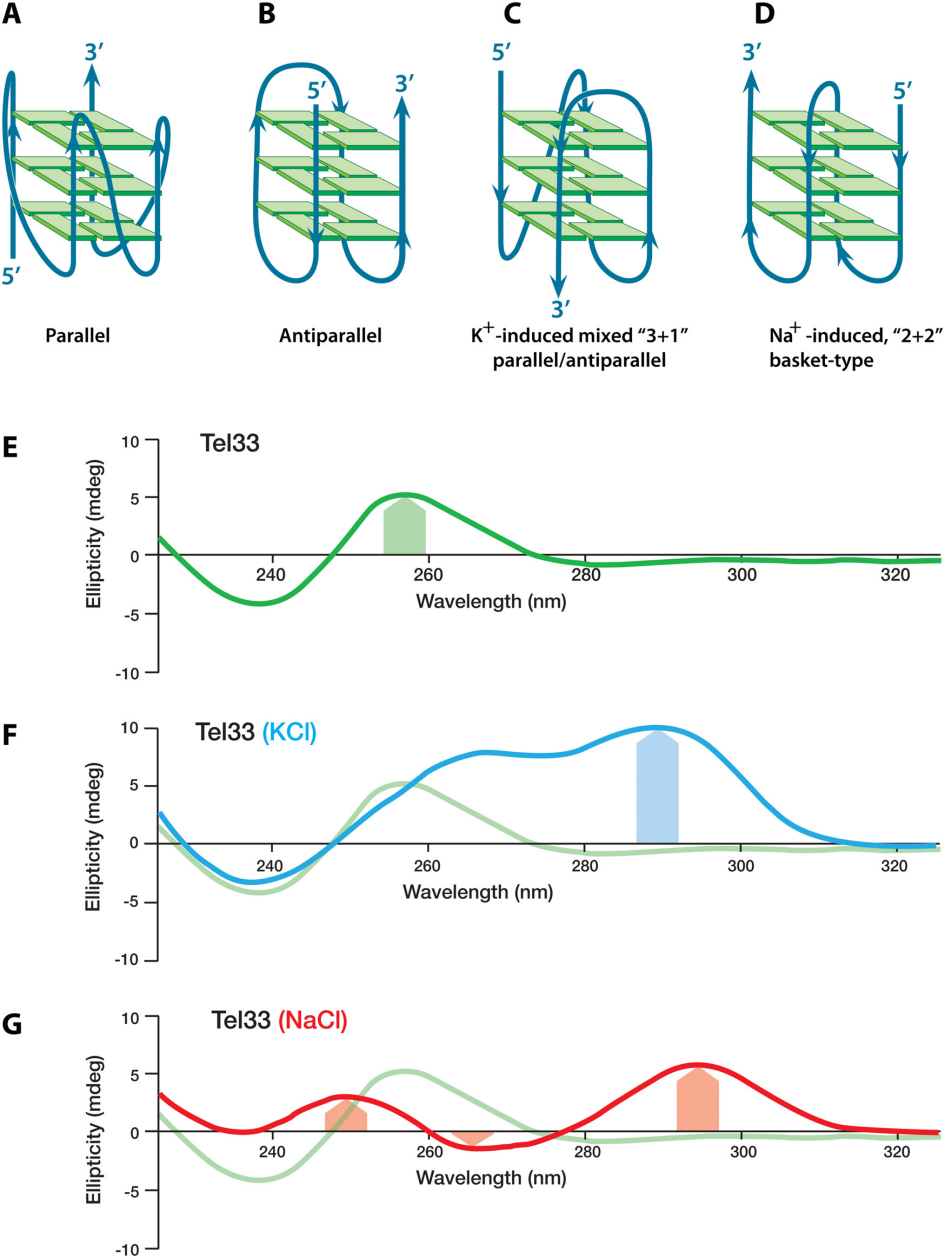
Telomeres exhibit cation-dependent, pleomorphic, unimolecular G4-DNA structures and produce characteristic signals on circular dichroism spectropolarimetry. Schematic depiction of (**A**) parallel-stranded, (**B**) antiparallel-stranded, (**C**) K^+-^induced telomeric mixed “3+1” parallel/antiparallel, and (**D**) Na^+^-induced telomeric basket-type “2+2” parallel/antiparallel G4-DNA structures. (**E**) CD spectrum of telomeric DNA in water without G4-structure shown in green. (**F**) CD spectrum of a telomeric K^+^-induced “3+1” parallel/antiparallel G4-structure with hallmark of 290 nm peak shown in blue. (**G**) CD spectrum of a telomeric Na^+^-induced basket type “2+2” parallel/antiparallel G4-structure with hallmark peaks at 250 nm and 295 nm with trough at 265 nm shown in red.

The repeated run of three guanines in the telomere represents the highest genomic concentration of DNA capable of forming G-quadruplex (G4-DNA or G4-structures). As demonstrated *in vitro*, the single-stranded G-rich overhang sequence of the telomere assumes a structure of intramolecular G4-structures in physiological salt conditions [9,10,11]. G4-structures can form in DNA and RNA via Hoogsteen bonding interactions within guanine-rich regions of the genome. Vertical stacking of Hoogsteen-bonded guanine tetrads and coordinate bonding with monovalent cations (i.e. K^+^, Na^+^) within the central cavity contribute to the remarkable thermal stability of G4-structures [12,13]. Bioinformatics studies suggest that the human genome contains >375,000 “potential G4-forming motifs” (PG4) [14,15]. The G4-structure adopted by telomeric DNA is thought to constitute a component of the protective telomeric cap possibly providing a default protective structure when specific DNA binding proteins are absent [16,17,18,19]. The degree of structural variation of telomeric G4-structures is high, as they are capable of forming tetramolecular, bimolecular, or unimolecular structures. Furthermore, unimolecular structures can adopt various intramolecular strand orientations including parallel, antiparallel, or mixed orientations as shown in Fig 1A–1D [13,20]. These structures produce characteristic spectra determined by circular dichroism spectropolarimetry (Fig 1E–1F).

The specific conformation adopted by telomeric G4-DNA has been observed *in vitro* as dependent upon the cationic environment, the number of telomeric repeats, flanking sequences, and DNA concentrations [21]. In K^+^-containing solutions, Tel22, a 22mer of the human telomeric sequence, forms unimolecular G4-structures with mixed parallel/antiparallel strand orientations (Fig 1C) [9,10,11]. These telomeric G4-structures contain three runs of guanines oriented in one direction and the fourth run oriented in the opposite direction, and is referred to as the “3+1” topology [9,10,22,23,24]. In solutions where Na^+^ is the only monovalent cation, Tel22 forms a basket-type G4-structure with two antiparallel strands adjacent to two parallel strands (“2+2” topology) (Fig 1D). Parallel versus antiparallel structural variation have known consequences on enzyme activity targeting these structures. For example, antiparallel Na^+^-stabilized telomere G4-sequences are readily extended by *Tetrahymena* telomerase, whereas K^+^-stabilized mixed-orientation structures are less-readily extended [25].

G4-DNA has unusually high thermodynamic stability, which is expected to affect telomere processing, such as inhibiting telomere degradation or extension. Therefore, it is likely that G4-DNA resolving enzymes accompany telomeric DNA replication. Direct evidence for the presence and resolution of G4-structures during replication has been demonstrated within the telomeres of ciliates *in vivo* [15,26], and recent work has quantitatively visualized G4-structures at the telomere in mammalian cells [27]. Since dynamic responsive control of telomere G4- structures is expected in cells, a number of candidate human proteins have been identified to promote formation or resolution of these structures *in vitro* and may contribute to that function *in vivo*: Topo I [28], GQN1 [29], BLM[30], WRN [31], FANCJ [32], hnRNP A1 [33], hnRNP D [34], Pot1[19], hnRNAP A2* [35], hPif1[36], ChlR1/DDX11 [37], and G4R1 [38,39,40,41,42].

G4 Resolvase 1 (G4R1), (also known as RHAU and the DHX36 gene product) is a member of the DEAH-box family of RNA helicases that has been identified as a modulator of an AU-rich element involved in mRNA metabolism [43], and our laboratory identified it as the major G4-DNA resolvase found in HeLa cells lysates [39,42]. G4R1 binds to parallel unimolecular G4-DNA with the tightest reported Kd for any G4-binding protein and unwinds this structure in an ATPase-dependent and non-processive manner [40,44]. It has been shown to have similar capacity to bind and resolve G4-RNA as well [38,39]. G4R1 is responsible for >60% of the tetramolecular G4-resolving activity in HeLa cell lysates [42], and the homozygous mouse knockout is embryonic lethal [45].

Recently, G4R1 has been shown to play a role in telomerase/telomere biology [38,41,46].

G4R1 down-regulation decreases levels of fully matured human telomerase RNA (hTR), and G4R1 appears to physically associate with the telomerase holoenzyme via the G4-forming sequence located at the 5’ end of hTR. If the hTR G4-sequence is deleted or mutated to prevent G4-formation, the association of G4R1 with telomerase is lost [38,41,46]. Furthermore, G4R1 unwinds hTR G4-structures, which appears to affect the assembly of the mature telomerase template RNA sequence and increases P1-helix formation *in vitro* [38]. Loss of the P1-helix is associated with reduced fidelity of telomerase [47]. The dual activity of G4R1 on G4-DNA and G4-RNA, and its association with the telomerase holoenzyme, could also give G4R1 access for activity upon the G4-DNA of telomeres. Thus far, down-regulation of G4R1 levels in 293T cells has shown a transient but significant change in average telomere lengths [38]; however, it is not known if G4R1 is capable of binding to telomeric G4-DNA structures.

In this study, we demonstrate that G4R1 is capable of tightly binding pleomorphic states of telomeric DNA including the 3+1 and 2+2 telomeric G4-structures, with a ~7-fold preference for the 3+1 topology. Thus, G4R1 shows a pan-specificity to G4-DNA. In the context of the telomeric sequence, we utilize systematic mutations to determine the length, sequence, and structural requirements sufficient for tight and complete G4R1-telomeric binding, thereby showing that the presence of G4-structure and a guanine-containing 3’-tail are sufficient for tight binding. These data are compatible with a model in which G4R1 modulates telomeric G4- structures that form with a free 3’ tail. This kind of structure could occur at the 3’ telomere end after it is displaced from a T loop by a replication fork.

## Materials and Methods

### Circular dichroism spectropolarimetry

Circular dichroism (CD) experiments were performed as previously described [40].

Briefly, oligonucleotides were purchased from Integrated DNA Technologies with or without the addition of a 5’-phosphate group and were analyzed on an AVIV Model 202 CD spectrometer equipped with a thermoelectrically controlled cell holder. Ten μM solutions of DNA were prepared in 10 mM Tris-HCl, 1mM EDTA, pH 7.5 and in the absence or presence of 50 mM KCl or 50 mM NaCl. CD spectral measurements were recorded in the UV (200–325 nm) regions with 1 nm increments and an averaging time of 2 s at 25, 37, 50, 60, 80, and 95°C. The thermal stability of Tel33 G4-DNA was determined by recording the molar ellipticity at 285 nm across the temperature range of 37–95°C, with 2°C increments and 30 s equilibration time at each temperature setting. Statistical analysis was performed with GraphPad Prism 5 and graphs were smoothed using LOWESS (20 point smoothing window).

### G4-DNA formation and quantification

Oligonucleotide concentrations were determined as previously described [40] by absorbance at 260 nm using a Smart Spec 3000 UV spectrometer (Bio Rad). The molar extinction coefficient was calculated using the instrument’s software after inputting the base composition of each oligonucleotide. Oligonucleotides were formed into unimolecular G4-DNA using a previously described procedure [40]. Briefly, oligonucleotides were diluted to a concentration of 0.5 mM with 10 mM Tris-HCl, 1 mM EDTA, pH 7.5 and incubated in a thermocycler (Eppendorf epGradient S) at 98°C for 10 min, then held at 80°C. Immediately the tube was opened and salt solutions were added to a final concentration of 50 mM (KCl or NaCl or 50 mM KCl/10 mM NaCl depending on the experiment). The tubes were closed and allowed to slowly return to 25°C and then stored at -20°C. Tetramolecular Z33 G4-DNA substrates were formed as previously described [38] and prepared in salt solutions to a final concentration of 50 mM (KCl or NaCl) or 50 mM KCl/10 mM NaCl.

### 5’-[32P]-end labeling of DNA

Unlabeled oligonucleotides were purchased from Integrated DNA Technologies. 5’-[32P]-end-labeled oligonucleotides were obtained as previously described [40]. Briefly, unlabeled oligonucleotides were annealed as described above, followed by 0.5 h incubation at 37°C with T4 polynucleotide kinase (Promega Corporation) and [γ-32P] ATP (Perkin-Elmer), according to manufacturer’s instructions. Following the labeling reaction, oligonucleotides were purified with a MicroSpin G25 column (GE Healthcare) equilibrated with TE (10 mM Tris, 1 mM EDTA, pH 7.5) buffer and stored at -20°C.

### Gel mobility shift assay and apparent K_d_ determination for G4R1-oligonucleotide complexes.

We have previously described the isolation [38,42] and quantification [40] of active recombinant G4R1 from Rosetta 2 cells. The apparent K_d_ was estimated using gel mobility shift assays (GMSA) as previously described [40]. Briefly, recombinant G4R1 at concentrations of 10–500 pM was incubated with 1 pM 5’-[32P]-end-labeled G4-DNA in Res buffer (50 mM KCl, 10 mM NaCl, 3 mM MgCl_2_, 50 mM Tris acetate, pH 7.8, 70 mM glycine, 0.012% bovine lactalbumin, 10% glycerol) with 10 mM EDTA at 37°C for 0.5 or 24 h. Binding reactions were loaded onto 10% non-denaturing polyacrylamide gels and electrophoresis was performed at 70 V for 15 h in a cold room (7°C). Gels were imaged on a Typhoon 9210 Imager (GE Healthcare), and band densities were analyzed using Multi Gauge software (Fuji). The apparent K_d_ was derived from the Scatchard equation using SigmaPlot 11.0 software to perform direct curve- fitting analysis. For binding experiments in which the monovalent cation composition was varied, recombinant G4R1 at concentrations of 50–500 pM was incubated with 1 pM 5’-[32P]-end labeled G4-DNA and Res buffer with either 50 mM KCl/10 mM NaCl, 50 mM KCl or 50 mM NaCl. Analysis was performed as described above.

## Results

### G4R1 binds to Tel30 and Tel33, but not to Tel22 or Tel26

**Fig 2:**
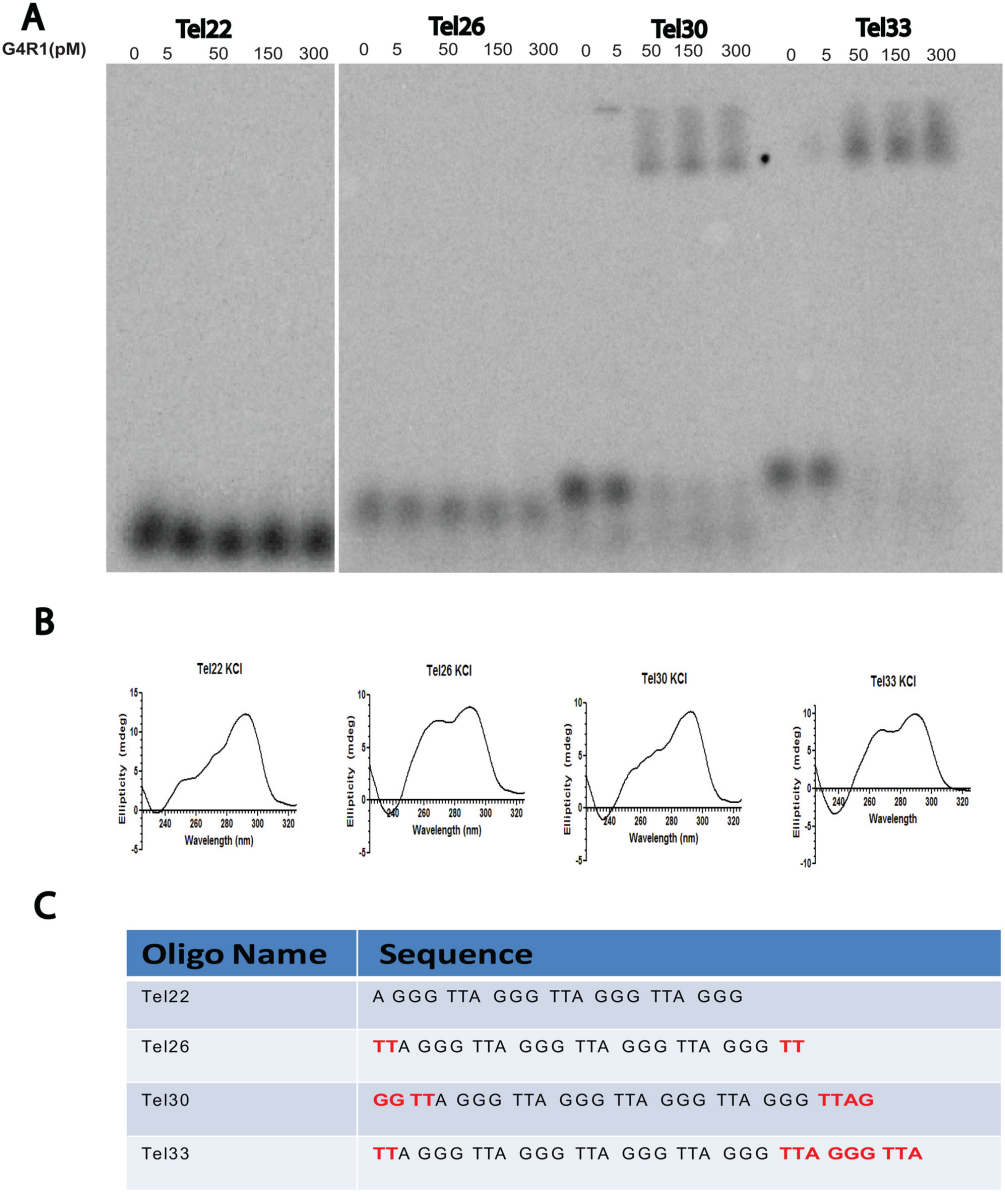
Tight G4R1 binding to the telomeric repeat sequence requires more than the minimal G4-structure found in Tel22. (**A**) Gel mobility shift assay (GMSA) with purified recombinant G4R1 and ^32^P-end-labeled telomeric G4-DNA sequences (left to right) Tel22, Tel26, Tel30, and Tel33 with indicated G4R1 concentrations. (**B**) CD wavelength spectra in 50 mM KCl of telomeric G4-DNA sequences (left to right) Tel22, Tel26, Tel30, and Tel33. (**C**) DNA oligonucleotide sequences used in Fig 2A and 2B.

We had previously demonstrated that a number of unimolecular parallel G4-DNA sequences tightly bind G4R1 using GMSA [40,48]. In the telomeric context, we first examined binding affinities of the well-characterized minimal G4-structure having just four runs of three guanines that has been shown in potassium to form a 3+1 quadruplex structure (Fig 1C) [9,10,11,21,22]. This minimal telomeric quadruplex showed no detectable binding to G4R1 (Fig 2A), although CD spectra showed that quadruplex structure is indeed forming under our binding conditions (Fig 2B). Our previous work had suggested that G4R1 requires a 3’-tail for tetramolecular G4-DNA resolution (S2 Fig). Therefore, we designed a series of oligomers that extended Tel22 with native telomeric sequences so that a 3’tail would exist, and termed the oligos Tel26, Tel30, and Tel33 (Fig 2C). Tel26 contains the Tel22 sequence with the addition of two native thymines on the 3’ and 5’-ends; however, G4R1 did not significantly bind to this structured sequence (Fig 2A). Interestingly, G4R1 bound to a 30mer and 33mer of the native human telomere sequence with affinities similar to that of previously reported G4-sequences (Fig 2A) [39,40,42,48], and with Tel33 demonstrating the most complete binding.

We compared the CD structural data of these sequences to determine whether aspects of CD spectrum were predicting G4R1-binding. Tel22, Tel26, Tel30, and Tel33 all displayed CD spectral patterns indicative of G4-structures with mixed parallel/antiparallel strand orientation; however, the distribution of peak signals at 263 nm and 290 nm varied between sequences potentially suggesting differing parallel (~263 nm) and antiparallel CD signal (~290 nm) contributions varied in distribution between the sequences. Tel22 and Tel30 were most similar to each other with major peaks around 290 nm and a shorter “shoulder” peak at 260 nm (Fig 2B). Tel26 and Tel33 both exhibited a more equal distribution between 260 nm and 290 nm peaks (Fig 2B). These variations existed in all CD spectra, and all had the hallmark 290 nm peak without a 250 nm peak (Fig 1F) indicating G4-DNA was forming in each structure in the “3+1” topology. G4R1 binding to telomeric DNA has very specific binding requirements and our data suggest that quadruplex CD spectral data alone do not accurately predict binding affinity because G4-structure alone is not sufficient for telomeric binding by G4R1. It was also of interest to us that binding ability appeared coincident to a fifth run of guanines being present in Tel30 and Tel33 oligomers. In other experiments (data not shown), we had determined that the first G in Tel30 was critical for binding, suggesting the importance of the fifth run of guanines (data not shown). Since runs of two guanines can form a stable G4-structure, Tel30 can form a 3’ G4-structure with a 5’ unstructured tail. Tel33 can form a similar structure with runs of three guanines participating in G4-structure. The results suggests that G4- structure alone (as in Tel22 and Tel26) is not sufficient for binding with G4R1, but a 3’ G4-structure with a 5’ tail (as in Tel30 and Tel33) may be sufficient for tight binding.

### G4R1 differentially binds to K^+^-induced and Na^+^-induced Tel33 G4-structures

Ionic environments can be critical determinants of G4-topology [20]. In this regard, it has been shown that the telomeric DNA repeat forms a 3+1 parallel/antiparallel G4-structure (as in Fig 1C) in K+-containing solutions, and a primarily basket-type G4-structure, the 2+2 antiparallel G4-structure, in Na^+^-containing solutions (as in Fig 1D) [49]. We sought to determine if G4R1 could recognize these two structures in Tel33.

**Fig 3:**
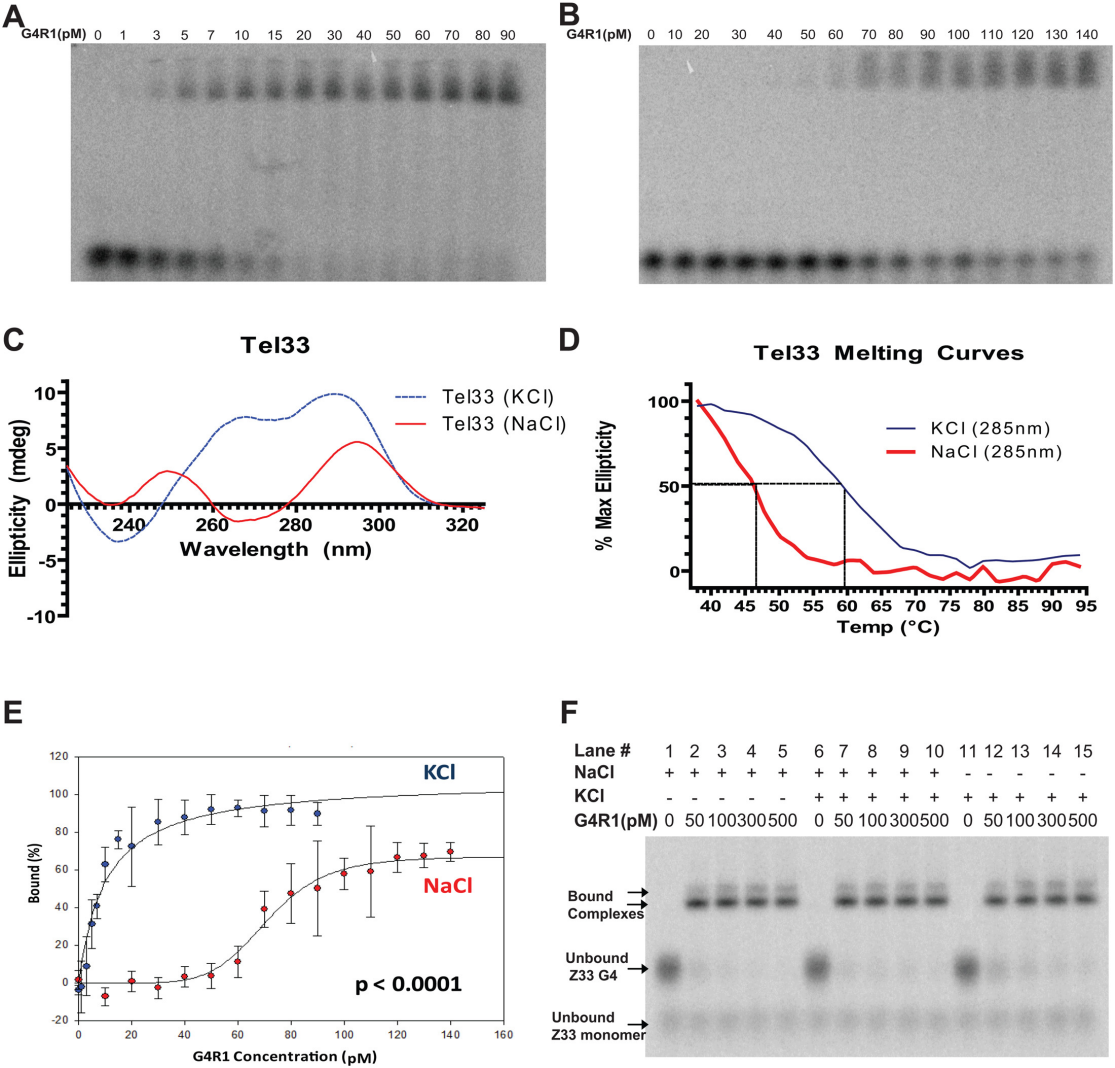
G4R1 tightly binds K^+^-induced telomeric mixed “3+1” parallel/antiparallel and Na^+-^induced telomeric basket-type “2+2” parallel/antiparallel G4-DNA structures, but its affinity for the K^+^-induced structure is greater. (**A**) Representative image of a GMSA with indicated concentrations of purified recombinant G4R1 and ^32^P-end-labeled Tel33 G4-DNA as substrate, incubated for 24 h in 50 mM KCl buffer. (**B**) Representative image of a GMSA with indicated concentrations of purified recombinant G4R1 and ^32^P-end-labeled Tel33 G4-DNA as substrate incubated for 24 h in 50 mM NaCl buffer. (**C**) CD wavelength spectra of Tel33 in 50 mM KCl and 50 mM NaCl. (**D**) CD melting curves determined by plotting the maximal ellipticity (mdeg) of KCl-formed Tel33 and NaCl-formed Tel33 at 285 nm versus temperature (°C). (**E**) Apparent K_d_ determination of G4R1 for K^+^-induced Tel33 G4-structure (blue) and Na^+^-induced Tel33 G4- structure (red), quantified from Fig 3A and 3B respectively (n = 3). (**F**) GMSA with indicated concentrations of purified recombinant G4R1 incubated for 24 h with ^32^P-end-labeled tetramolecular Z33 G4-DNA in 50 mM NaCl (lanes 1–5), 50 mM KCl/10 mM NaCl (lanes 6–10) or 50 mM KCl (lanes 11–15).

Tight binding of G4R1 to the K^+^-induced Tel33 G4-sequence was observed, with an apparent Kd of 10 ±1.9 pM (n = 3) (Fig 3A and 3E). G4R1 bound to the Na^+^-induced Tel33 G4-structure with an apparent Kd of 71 ± 2.2 pM (n = 3) (Fig 3B and 3E). In order to rule out the possibility of an inhibitory effect of Na^+^ ions on G4R1 binding, we assessed the binding of G4R1 to a tetramolecular G4-substrate (Z33) whose structure is formed in Na^+^ but is maintained as a stable, parallel, tetramolecular G4-structure under both monovalent salt conditions. G4R1 bound equally well to tetramolecular G4-DNA in the presence of NaCl, KCl/NaCl, and KCl buffers (Fig 3F), thus ruling out a potential inhibitory effect of sodium ions on the G4R1/G4- DNA binding interaction or enzyme conformation, confirming our previous observations [39].

A Tel33 CD spectrum is indicative of the 3+1 parallel/antiparallel G4-structure in 50 mM KCl-containing buffer (Fig 3C). Furthermore, the thermal stability of the molar ellipticity peaks at 263 nm and 285 nm was monovalent cation-dependent and did not form in the absence of K^+^ (S1A Fig).). The presence of a CD spectrum characteristic of G4-structure and its high monovalent cation-dependence are evidence of the presence of formed G4-DNA structure. When we formed Tel33 in a pure Na^+^ environment, the CD-spectra were indicative of antiparallel 2+2 G4-structure [9] consisting of a large peak of positive molar ellipticity at ~285 nm, a small negative peak at ~265 nm and a small positive peak at 245 nm (Fig 3C). The Tm of each Tel33 structure was determined by measuring the temperature at which 50% of the molar ellipticity at 285 nm was lost. The K^+^-induced and Na^+^-induced structures were thermally stable, exhibiting Tm of 59.6°C and 46.7°C at 285 nm, respectively (Fig 3D). Taken together, these data demonstrate that G4R1 binds remarkably tightly to the mixed parallel/antiparallel Tel33 G4-structure with an affinity similar to what we previously reported for parallel-stranded G4-structures [39]. G4R1 also has strong affinity for Na^+^-induced antiparallel 2+2 Tel33 G4-structure albeit ~7 fold less.

### Single guanine to adenine mutations in the Tel33 sequence dramatically affect G4R1 binding but not the CD structural spectrum

**Fig 4:**
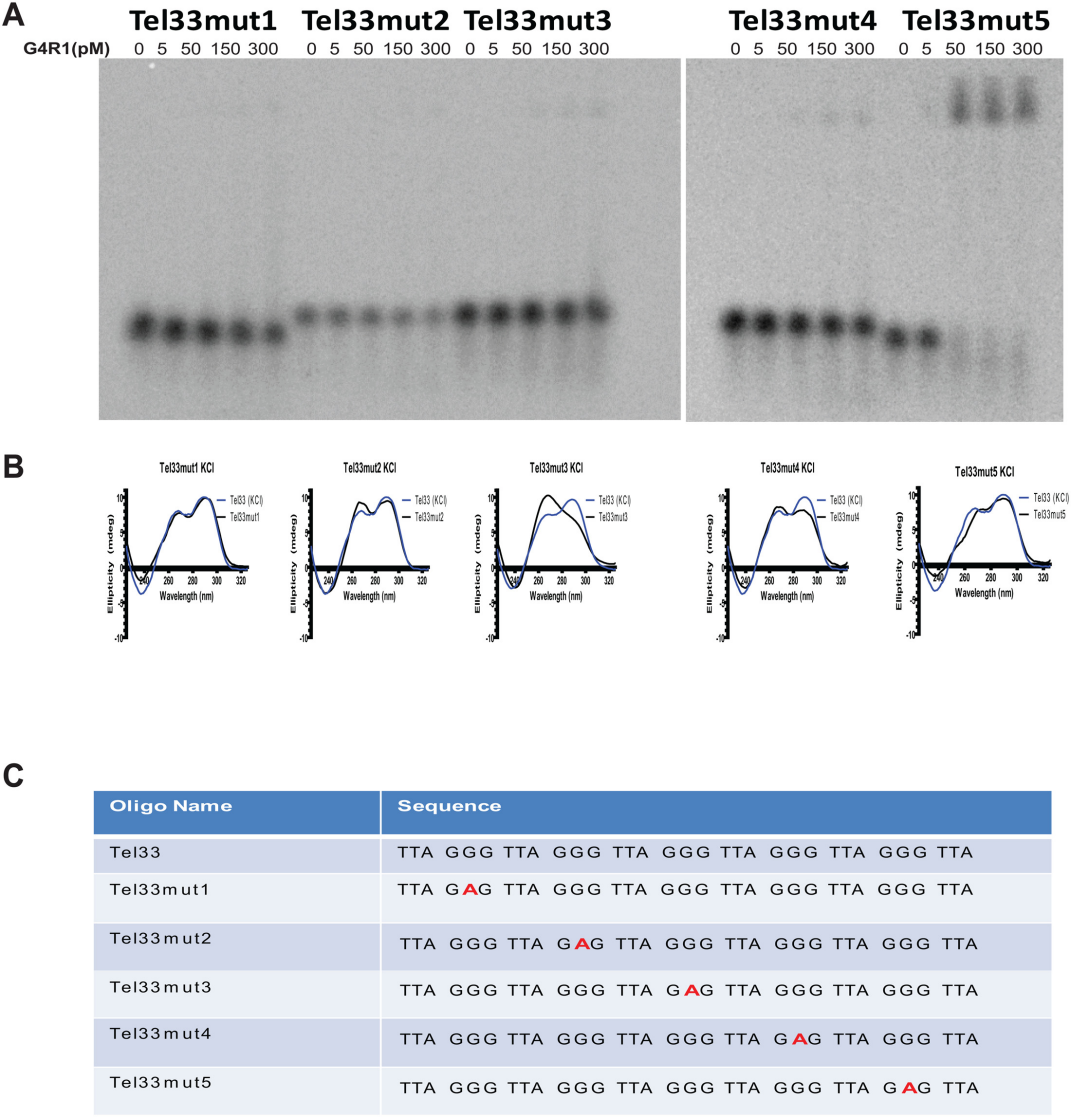
Single targeted mutations expected to change guanine run participation in G4-DNA structure concomitantly disrupt G4R1 binding with the exception of the central guanine at the most 3’ guanine run. (**A**) GMSA with indicated concentrations of purified recombinant G4R1 and ^32^P-end-labeled mutant telomeric G4-DNA sequences (left to right) Tel33mut1, Tel33mut2, Tel33mut3, Tel33mut4, and Tel33mut5. (**B**) CD wavelength spectrum in 50 mM KCl of mutant telomeric G4-DNA sequences (left to right) Tel33mut1, Tel33mut2, Tel33mut3, Tel33mut4, and Tel33mut5 (black traces), each overlaid with spectra ofTel33 in KCl (blue traces). (**C**) DNAoligonucleotide sequences used in Fig 4A and 4B.

Given the substantial differences of G4R1 binding affinity for Tel22, Tel26, Tel30, and Tel33 sequences, we sought to determine the critical requirements for tight G4R1 binding to Tel33 in potassium. To do this, we made a series of five mutants in which the central guanine in one of the five tracts of guanines was successively mutated to adenine. It should be noted that such a mutation in the central guanine of three continuous guanines will prevent participation of that run of guanines in the G4-stucture. The five mutants were termed Tel33mut1, Tel33mut2, Tel33mut3, Tel33mut4, and Tel33mut5 (Fig 4C), with Tel33mut1 having the 5’ most guanine tract mutated and Tel33mut5 having the 3’ most guanine tract mutated. Since base context is of importance, it is debatable how large a loop area can be expanded before four runs of guanines are unlikely to participate in a G4-DNA structure, although stable loop length has been shown to be clearly longer than a limit of 7 bases [50]. As shown empirically by our CD data, Tel33mut2, Tel33mut3, and Tel33mut4 appear to have nine base loops and yet form stable G4-DNA structures. The evidence for this is twofold: first, the CD spectral data are indicative of G4-structure (Fig 4B) (discussed below); second, the monomer spot on the gels of these oligomers is the same length yet migrates considerably higher for Tel33mut2, Tel33mut3, and Tel33mut4 suggesting each structure has a similar bulky loop, while the more compact structures of Tel33mut1 and Tel33mut5 migrate faster and also similarly (Fig 4A). GMSA analysis shows that the single-base mutations in Tel33mut1, Tel33mut2, Tel33mut3, and Tel33mut4 markedly decreased G4R1 binding (Fig 4A). However, Tel33mut5 retained similar binding affinity to G4R1 as that of Tel33 (Fig 4A).

Next, we measured the CD spectral data of these sequences to determine mutational effects on G4-DNA folding patterns in potassium. All CD spectra indicated G4-structure of the 3+1 type forms. Tel33mut1, Tel33mut2, and Tel33mut4, in which the guanine to adenine mutation markedly diminishedG4R1 binding (Fig 4A), showed no appreciable change in the CD spectrums compared to that of Tel33 (Fig 4B). Tel33mut3 displayed a spectrum similar to Tel33 but with a higher 263 nm peak and a lower 285 nm peak (Fig 4B). Tel33mut4 had a slightly lower 285 nm peak than Tel33. Tel33mut5 displayed a spectrum similar to that of the K+-induced Tel33 G4-structure and bound G4R1 with similar affinity. These data further support the idea that G4-structure alone is not sufficient to predict binding of G4R1 to telomeric DNA. Of key importance is that Tel33mut5 binds G4R1 tightly and completely. This suggests that a 5’ G4-structure and a 3’ unstructured tailare sufficient for binding. In the cases of all other mutants, since CD indicated that G4-structure was present, the most 3’ guanine run must participate in the G4-structure, limiting the 3’ tail to only three nucleotides: TTA. These results suggest that a G4-structure and a 3’ tail of TTA is not sufficient for binding; however, a G4-structure and an 11-base tail may be sufficient for binding.

### A free 3’ tail partially restores binding of G4R1 to Tel22

In studying the resolution of tetramolecular quadruplexes, we found that G4R1 requires a 3’ tail in order to effectively unwind tetramolecular G4-structures (S2 Fig).). These data also made us consider that tight G4R1 binding to telomeric G4-DNA might require a sufficient 3’ tail in addition to quadruplex structure. For example, G4-Tel22 would lack a 3’ tail completely, while Tel26 would have at most a two nucleotide 3’ tail. We reasoned that the increase of G4R1 binding to Tel30 compared to that of Tel22 or Tel26 was due to the addition of 5’ guanines (Fig 2A). These guanines could shift the G4-structure and allow the inclusion of the 5’-most runs of guanines in G4-DNA, thus freeing up the 3’-most runs of guanines to form an unstructured 3’ tail. Similarly, the Tel33 sequence with five runs of guanines would allow a dynamic equilibrium of G4-structures formed between the 5’and 3’ends. Furthermore, the mutation introduced into Tel33mut5 excludes the 3’-most run of guanines in G4-formation, thus shifting the G4-DNA structure to form with the 5’-most runs of guanines, probably leaving the 3’-end uninvolved in G4-formation. These data suggest that binding of G4R1 to telomere G4-DNA requires a 3’ tail.

**Fig 5:**
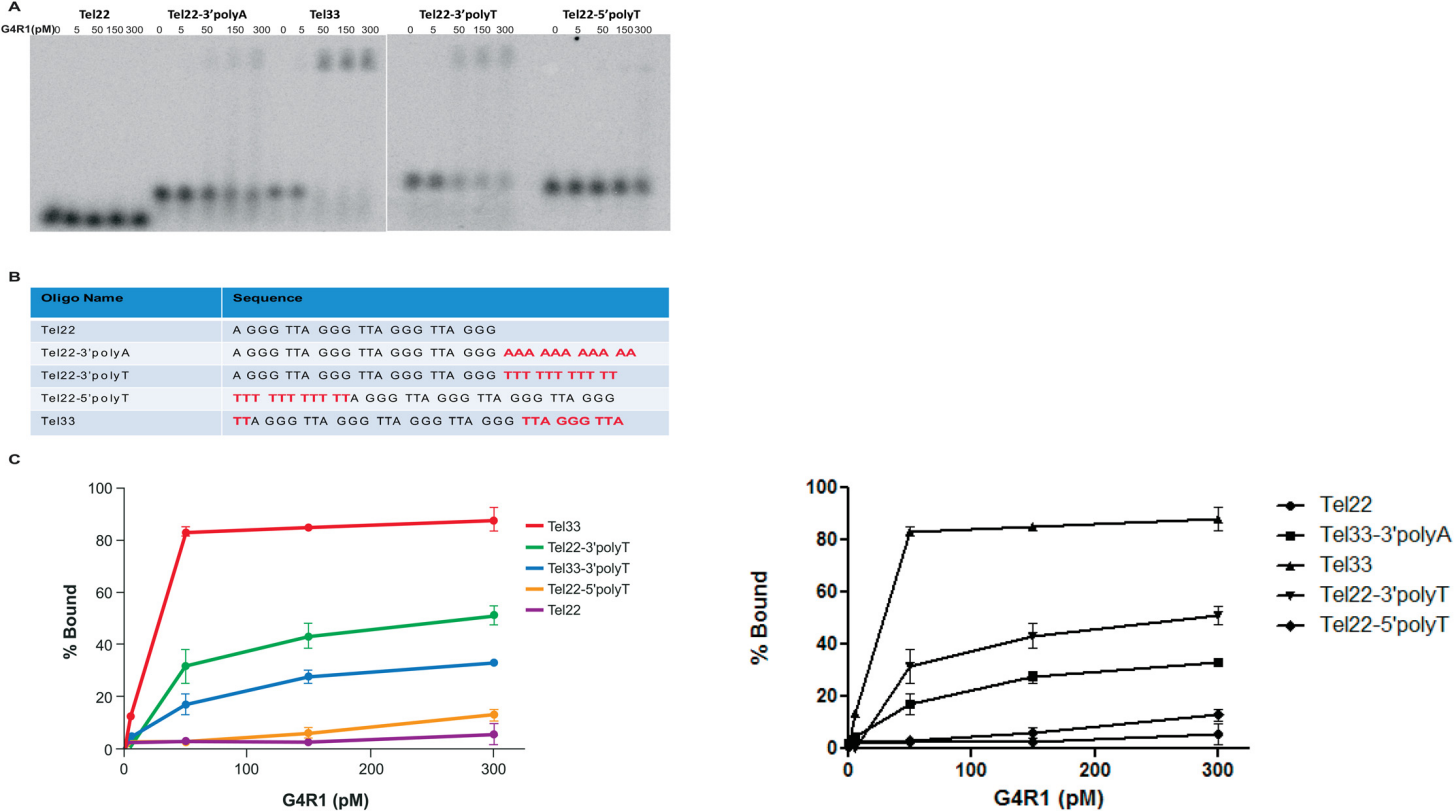
Adding an unstructured 3’-tail to Tel22 only partially restores binding to telomeric repeat sequences. (**A**) GMSA with indicated concentrations of purified recombinant G4R1 and ^32^P-end-labeled mutant telomeric G4-DNA sequences (left to right) Tel22, Tel22- 3’polyA, Tel33, Tel22-3’polyT, and Tel22-5’polyT. (**B**) DNA oligonucleotide sequences used in Fig 5A and 5B. (**C**) Graphical depiction of GMSAs quantified from three experiments including Fig 5A.

To test the hypothesis that 5’ quadruplex with a 3’ unstructured tail is sufficient for tight G4R1 binding, we designed Tel22-variants in which 11-nucleotide tails (3’ polyA, 3’ polyT, or 5’ polyT) were added to the core Tel22 sequence and these were named Tel22 3’polyA, Tel22 3’polyT, and Tel22 5’polyT, respectively (Fig 5B). GMSA binding affinities for each of these oligos were tested alongside Tel22 and Tel33, which were used as negative and positive controls respectively (Fig 5A), and binding affinities of three repeated experiments were subsequently quantified (Fig 5C). Tel22 showed no binding. Tel22 3’polyA showed partial binding at the higher enzyme concentrations (Fig 5A and 5C), while Tel22 3’polyT demonstrated improved binding affinity; these did not, however, approach the affinity seen for Tel33 (Fig 5A and 5C). Tel22 5’polyT had no ability to restore binding affinity to the Tel22 core (Fig 5A and 5C), which was expected because it would only have a 3’ G4-DNA structure without a 3’ free tail. Although binding affinities of Tel22 variants significantly increased with the addition of 3’ tails, particularly with the 3’polyT tail, binding affinity was significantly less than that observed for Tel33 (Fig 5A and 5C). These data suggest that polyA and polyT 3’ tails added to Tel22 improve affinity, but elements found in Tel33 and Tel33mut5 allow tighter binding to G4R1.

### G4R1 requires a 3’ tail containing a guanine that is flanked by adenine(s) for tight binding to telomeric G4-DNA

**Fig 6:**
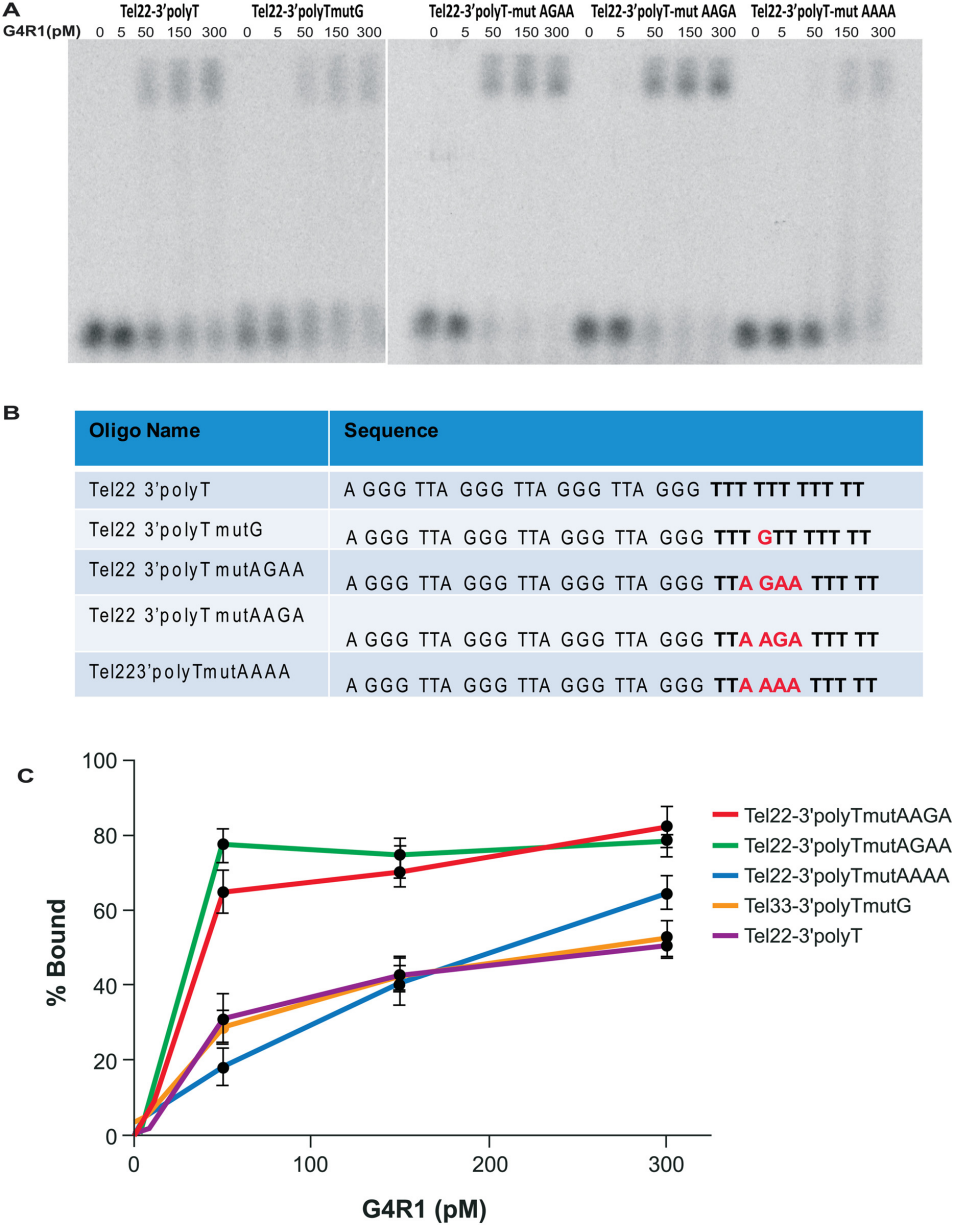
A guanine-containing 3’-tail with flanking adenines is sufficient to restore tight binding to telomeric G4-sequences by G4R1. (**A**) GMSA with indicated concentrations of purified recombinant G4R1 and ^32^P-end-labeled mutant telomeric G4-DNA sequences (left to right) Tel22-3’polyT, Tel22-3’polyTmutG, Tel22-3’polyT-mutAGAA, Tel22-3’polyT-mutAAGA, and Tel22-3’polyT-mutAAAA. (**B**) DNA oligonucleotide sequences used in Fig 6A. (**C**) Graphical depiction of GMSAs quantified from three experiments including Fig 6A.

Since adding 3’ poly A and polyT tails to Tel22 was not sufficient to completely restore tight binding of G4R1, we hypothesized that an additional sequence requirement within the 3’tail may be needed to facilitate binding. Because Tel33mut5 binds G4R1 with similar affinity to Tel33, we hypothesized that G4R1 requires a 3’ tail base change that breaks the redundancy of the tail, or perhaps a guanine was important in the 3’ tail. Therefore, we designed a set of mutants based on the Tel22 3’polyT sequence to test the 3’ binding requirements (Fig 6B). To determine if there is a requirement of guanine within the 3’ tail for tight binding of G4R1, we added a guanine to the 3’-polyT tail (Tel22- 3’polyTmutG). We found that G4R1 binds to Tel22-3’polyTmutG with similar affinity to Tel22-3’polyT (Fig 6A and 6C) suggesting that a guanine-containing 3’-polyT tail is not sufficient for complete binding.

In order to mimic the purine/pyrimidine content of a native telomere tail and break up tail redundancy, we synthesized a construct with four adenines within a polyT tail, termed Tel22- 3’polyT-mutAAAA. We found that G4R1 does not bind this sequence tightly and fully, suggesting that mimicking part of the purine/pyrimidine content within the polyT tail is not sufficient for complete binding.

Next, we tested oligomers in which one guanine was placed within the 3’polyT tail at different positions within flanking adenines. The objective was to add sequences into the tail that would more closely mimic a true telomere tail, but would not induce G4-formation. We termed these sequences Tel22-3’polyT-mutAAGA and Tel22-3’polyT-mutAGAA (Fig 6B). The addition of one guanine within the polyT tail, regardless of the tested position or flanking sequences in both oligomers, restored tight binding of G4R1 (Fig 6A) similar to that of Tel33 and Tel33mut5. These data show that a 5’ quadruplex and a substantial (~11nt) 3’ tail which also contains a guanine flanked by adenine(s) is sufficient for inducing highest binding affinity to G4R1.

### G4-structure is important for complete G4R1 binding to DNA

**Fig 7:**
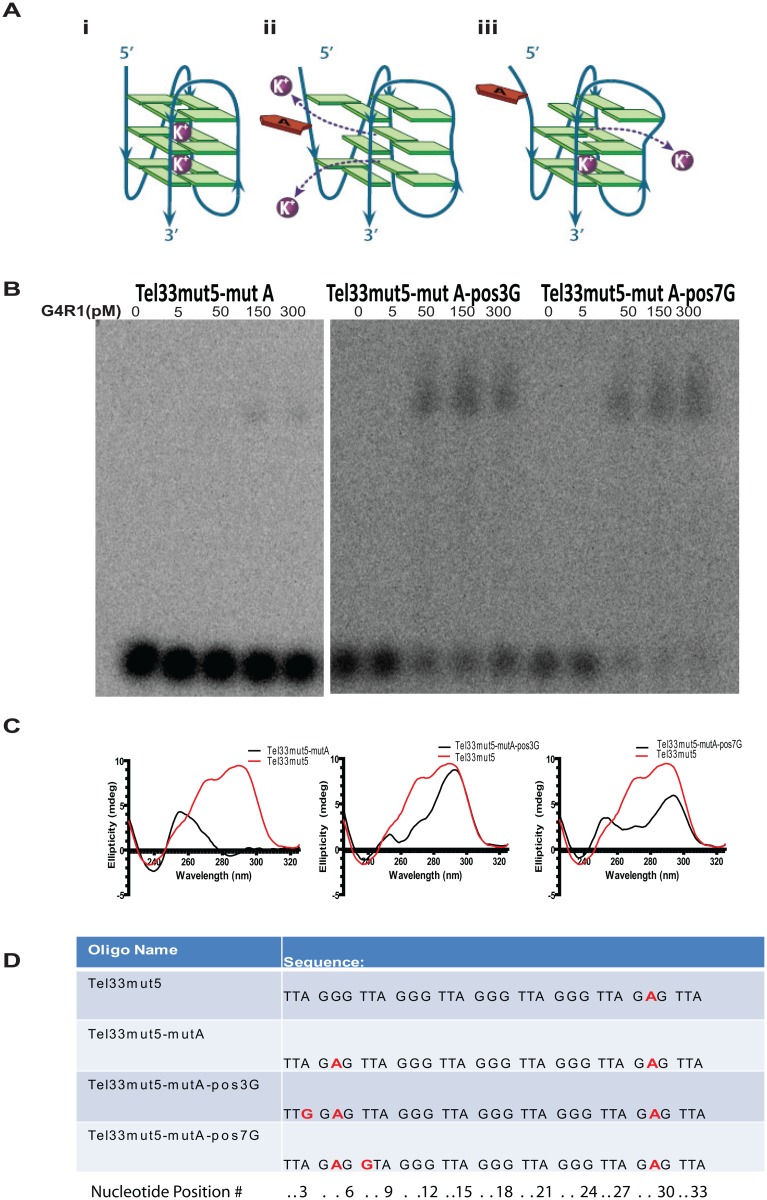
A single guanine mutation disrupts the 5’G4-DNA structure of Tel33mut5 and concomitantly disrupts G4R1 binding, but two secondary mutations that partially restore G4- DNA structure also restore binding. (**A**) Schematic depiction of a stable unimolecular G4-DNA structure with three guanine tetrads and two coordinate-bonded K^+^ ions (in purple) (i); G4-DNA structure is completely destabilized by the effects of a single G to A mutation at the central guanine of the 5’ most guanine run. The central guanine tetrad breaks apart and two coordinate bonded K^+^ ions (in purple) are lost (ii); Situations that restore two 5’ guanines in a row or mutations that keep two 5’guanines in a row intact can produce G4-DNA stabilized by two G- tetrads and a single-coordinate K+ ion (in purple). (**B**) GMSA with indicated concentrations of purified recombinant G4R1 and ^32^P-end-labeled mutant telomeric G4-DNA sequences (left to right) Tel33mut5-mutA, Tel33mut5-mutA-pos3G, and Tel33mut5-mutA-pos7G. (**C**) CD wavelength spectra in 50 mM KCl of mutant telomeric G4-DNA sequences (left to right) Tel33mut5-mutA, Tel33mut5-mutA-pos3G, and Tel33mut5-mutA-pos7G. (**D**) DNA oligonucleotide sequences used in Fig 7B and 7C.

For a protein capable of tightly binding telomeric DNA, such as G4R1, it is difficult to distinguish sequence binding requirements from potential G4-DNA structural requirements because sequence defines G4-structure. In the following experiment, we show that G4-DNA structure rather than telomere sequence is likely the key 5’ binding determinant. We did this by creating a single base sequence change in Tel33mut5 that ablates G4-DNA structure (and thus G4R1 binding as well), and then creating two different secondary mutated oligomers that rescue the G4-DNA structure to test if binding is recovered by these secondary mutations. The secondary mutations maintain the change that originally abolished binding, but allow us to test if binding can be restored by the secondary rescue mutations which restore G4-DNA structure. It should be noted that a single adenine mutation within any central guanine of the four runs of three guanines in a row is expected to fully destabilize a whole G4-structure as shown in Fig 7Aii.

Therefore, to inhibit G4-DNA binding, we designed a single base change of Tel33mut5 which contained a guanine to adenine mutation in the central guanine in the 5’ end (position 5) (See Fig 7D). This oligomer is expected to be unable to form stable G4-DNA. We termed this oligomer Tel33mut5-mutA, and CD analysis confirmed that G4-DNA structure was ablated as indicated by the substantially suppressed peaks at ~285 nm and ~263 nm (Fig 7C). The corresponding GMSA showed negligible binding to G4R1, even when the oligomer band was highly exposed in the gel (Fig 7B). This suggests that G4-structure is necessary for tight binding of G4R1.

Next, we created two oligomers that kept this original adenine mutation in Tel33mut5-mutA, but to each we added a single second mutation to create two guanines in a row that could restore the potential for a G4-structure to form. The resulting structures are expected to display renewed G4 CD signals, but of less amplitude, due to the formation of G4-structure within only two guanine tetrads rather than three participating in typical telomeric G4-DNA (see Fig 7A i versus iii). Tel33mut5-mutA-pos3G had an adenine mutated to guanine at the third nucleotide position, while Tel33mut5-mutA- pos7G had a thymine mutated to guanine at the seventh nucleotide position (Fig 7D). CD spectra confirmed that G4-structure is partially restored by the secondary point mutations (Fig 7C). The CD spectra demonstrated characteristic G4-peaks, albeit with shorter amplitudes probably due to having only two G-tetrads participate in G4-formation instead of three. When analyzed by GMSA, both oligomer mutations which restored significant G4-formation also restored significant binding for G4R1 (Fig 7B). In the case of Tel33mut5-mutA-pos7G, complete binding was restored. Taken together, the above data strongly suggest that G4-structure is the common requirement for binding and that sequence can be significantly changed from the canonical telomere repeat and maintain tight binding. Restoration of G4-structure correlates with binding. In addition, these results show that long runs of guanines alone in the absence of G4-structure cannot bind G4R1 tightly, as is the case for Tel33mut5-mutA, and that G4R1 can recognize quadruplex in a two- as well as a three-tetrad structure. These data strongly support the supposition that a 5’ G4-structure is necessary for G4R1 binding to the telomere sequence.

## Discussion

It is known that 85–90% of all cancers rely on the reverse transcriptase known as telomerase to maintain their telomere lengths, allowing them to divide indefinitely [6]. Telomere G4-structures have been shown to inhibit telomerase-catalyzed extension of the guanine-rich strands of telomeric DNA [18], making telomere G4-stabilizing ligand development an area of intense focus for cancer therapy drugs [51,52,53,54]. Considering that G4R1 has been shown to bind G4-DNA tightly and preferentially in comparison to Watson-Crick duplex-DNA, and recent studies suggest that G4R1 associates with the telomerase holoenzyme [38,41,46], we sought to determine the affinity of G4R1 specifically for telomeric G4-DNA structures.

In human cells, the single-stranded G-rich strand of the telomere is typically ca. 50–200 nt in length with 8–50 runs of three guanines [4]. Previous structural work on the human telomeric repeat has been predominantly studied using a 22mer repeat containing four runs of guanines (with the sequence of d[AGGG (TTAGGG)3]) known as Tel22, and it forms a 3+1 parallel/antiparallel structure in K^+^-solution [9,10,11,22,55]. Structural studies also suggest that telomeric DNA spanning five to seven repeats forms a similar topology. Our previous work has demonstrated that G4R1 binds to parallel unimolecular G4-DNA with remarkable affinity [40]. In this study, we demonstrated that in the context of telomeric DNA, G4-structure alone is not sufficient for binding, but that a 5’ G4-structure and a 3’ tail containing a guanine which is flanked by adenine(s) and within the context of a polyT tract, is sufficient for tight binding. If this requirement is met it appears that G4R1 can bind to 3+1 parallel/antiparallel G4-structures with similarly tight affinities as to parallel G4-structures [40], and with a ~7 fold lesser affinity to 2+2 antiparallel G4-structures. To our knowledge, G4R1 demonstrates the tightest reported affinity for telomeric G4-DNA of known human telomeric G4-binding proteins [39].

Our GMSA experiments demonstrated a ~7 fold difference in binding affinities of G4R1 for the K^+^- versus the Na^+^-induced Tel33 G4-structures. The fact that G4R1 effectively binds Na^+^-formed tetramolecular parallel DNA indicates that a G4-structure coordinately bonded with a Na+ core is an excellent binding substrate for G4R1. Thus, it is possible that the conversion of one of the three parallel strands into antiparallel orientation weakens the binding site for G4R1 in Na^+^-induced telomeric G4-DNA. Although our data suggest that G4R1 preferentially binds to parallel or 3+1 mixed G4-structures compared to 2+2 antiparallel structures, both structures display remarkably tight binding. This is compatible with a model in which G4R1 binds to a variety of G4-conformations and could act as a potential “pan” G4-resolvase.

Other human telomeric G4-binding helicases, such as BLM or WRN, demonstrate ATP- dependent helicase activity upon parallel tetramolecular telomere G4-DNA [5,16,30]. A recent publication shows that WRN has been shown to bind to a unimolecular telomere G4-structure formed in K^+^ (presumably a “3+1” structure) more tightly than does BLM [56]. WRN binds most tightly to a dimer telomere G4-structure formed in K^+^(a structure having two antiparallel strands) with an apparent Kd of 260 pM, whereas BLM did not bind to this structure [56]. BLM does bind to tetramolecular parallel DNA with an apparent Kd of 700 pM [52]; thus, it is possible that BLM is demonstrating a trend of preferential binding to parallel G4-DNA. The human POT1 protein is thought to recognize antiparallel unimolecular G4 telomeric structures and passively trap the associated sequence in an unwound form [8,19]. Recent observations of POT1 binding utilizing FRET suggest that POT1 has a slight binding preference to the Na^+^-induced telomere G4-structure over the K^+^-induced structure [57]. The proteins hnRNP A1 and hnRNP D are thought to passively resolve dimeric and antiparallel/parallel telomeric G4-structures, respectively [8,33,34], while hnRNP A2* is able to actively unfold a presumably 3+1 mixed structure [34]. Our current data, coupled with previously published work [40], suggest that G4R1 is a G4-helicase that can bind to an array of G4-structures including parallel tetramolecular [39], and parallel [40], mixed, and antiparallel unimolecular G4-structures.

The nucleus of a cell contains a K^+^ ion concentration substantially higher than the Na^+^ ion concentration. Thus, the telomeric DNA sequence is likely to adopt a mixed parallel/antiparallel G4-structure *in vivo*. We speculate that G4R1 could potentially have two actions on telomeric G4-DNA, depending on the availability of ATP and Mg2+, which G4R1 requires for resolution of G4-structures [38,39,40,41,48]. Under resolving conditions (+ATP, Mg2+), G4R1 would bind telomeric G4-structures and unwind them into unstructured single strands to allow telomerase action. Under non-resolving conditions, G4R1 would bind G4- structures and eventually “trap” the telomere in this G4-conformation, thus inhibiting telomerase. Recently, G4R1 has been shown to be a positive regulator of telomere length in 293T and HeLa cell lines [38], compatible with a model that G4R1 is acting under resolving conditions at the telomere.

**Fig 8:**
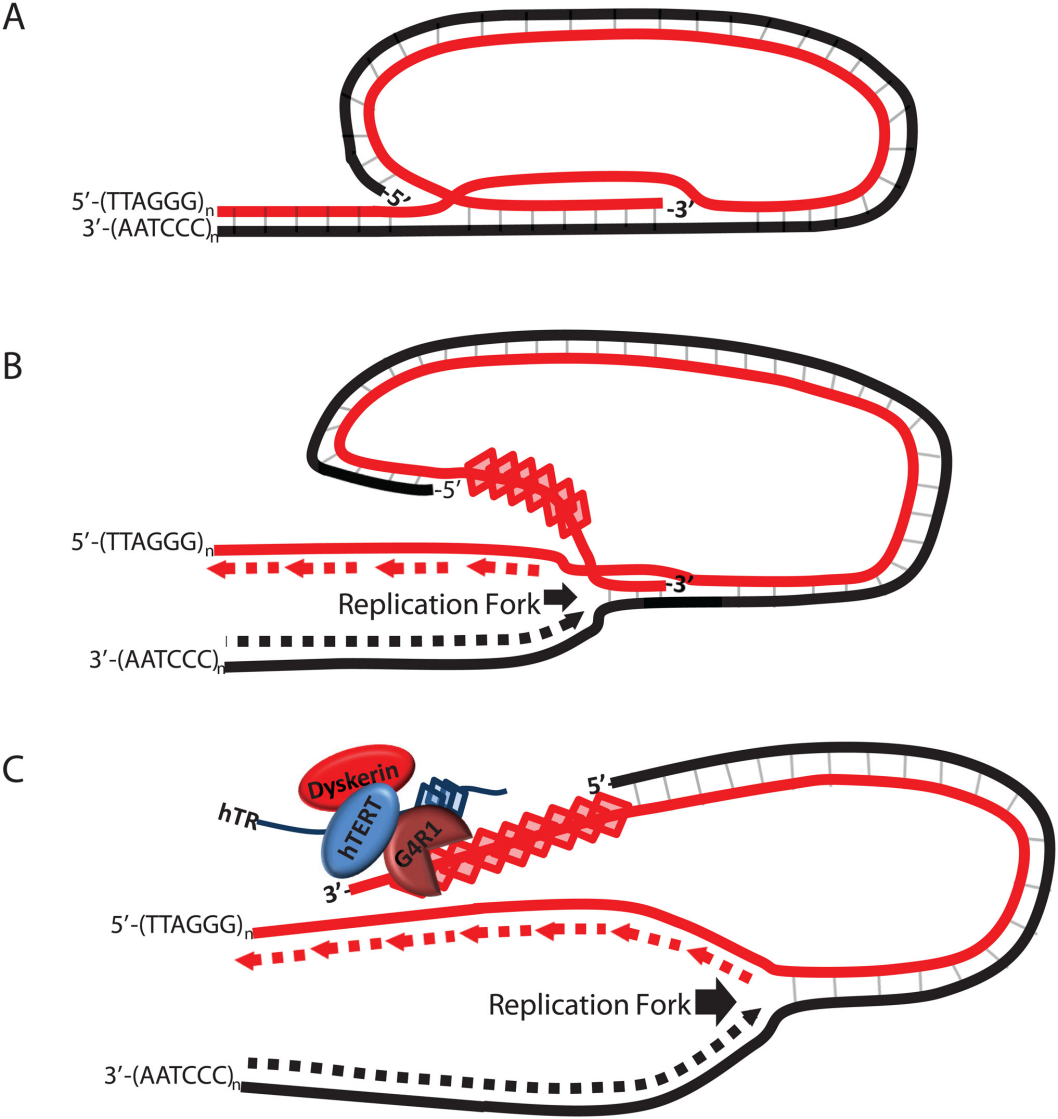
Schematic depiction of: (A) telomere in a T-loop; (B) T-loop with an incoming replication fork allowing G4-DNA to form at the 5’ side of the 3’ overhang; (C) displaced unstructured 3’ end plus quadruplex region that would allow tight binding by G4R1. G4R1 is depicted associated with the telomerase holoenzyme which also includes: hTR (TERC), hTERT, and Dyskerin.

Our data demonstrating G4R1’s high affinity for telomeric G4-structures *in vitro* suggest a potential role in unwinding these inhibitory G4-structures *in vivo*. G4-structures at the telomere are thought to inhibit telomerase [18,58]. We found that a 3’ guanine-containing tail is required for tight binding of G4R1 to telomere G4-structures. We also found that the 3’ tail was not only needed for binding telomeric DNA, but was also necessary for G4-resolution by utilizing a tetramolecular unwinding assay (S2 Fig).). These data suggest that if G4-structures were to form at the very 3’ end of the telomeres, G4R1 would be unable to bind these structures due to the lack of a sufficient 3’ tail. Similarly, telomerase requires at least an 8 nt 3’ tail to extend the telomeres [59] and, accordingly, G4-structure at the very 3’ end of the telomere blocks telomerase access. Although how this block is relieved is not fully understood, it has been suggested that proteins such as hnRNPA2* may play a role in this process through the disruption of telomeric G4-structures which lack free 3’ tails. hnRNPA2* can bind (K_d_ = 11.05 nM) and unwind the 4mer telomere repeat (GGGTTA)3GGG G4-structure which lacks a free 3’ tail [35]. G4R1 does not bind to the 4mer Tel22 telomeric repeat G4-structure, but does bind with remarkable affinity (apparent Kd = 10 pM) to telomeric G4-sequences which maintain a guanine- containing 3’ tail. One hypothetical model that could assign a direct role for G4R1 at the telomere would be to consider that the 3’ single-stranded overhang could frequently have runs of guanines that are not a multiple of four. If we consider that during DNA replication, T loops are displaced by an incoming internal 5’ replication fork, the last DNA removed from the T loop would be the very 3’ end guanine run as shown in Fig 8. For a 3’ single stranded region with guanine runs that are not of a multiple of four, the 3’ end would be left unstructured. This is because the G4-DNA would be forming 5’ to the end of the 3’ overhang as the T loop was peeled apart (Fig 8). G4R1 associated with telomerase would then tenaciously bind to the 3’ unstructured tail at the first G4-structure allowing telomerase to find the free 3’end to begin extension. If the 3’end is a multiple of four and forms into a G4-structure, then hnRNPA2* may provide telomerase access by unwinding G4-structures at the very 3’ end. Another interesting model that would provide a role for G4R1 to enable telomerase action involves the possibility that 3’ telomere DNA could be protected with a bimolecular quadruplex incorporating TERRA RNA. G4R1 has unique dual specificity at resolving RNA and DNA G4-structures that would be ideal for that job if such hybrid dimeric structures protect the telomere.

## Supporting Information

S1 Fig Tel33 rapidly forms and maintains a mixed parallel/antiparallel structure in K^+^-containing solution. (A) CD wavelength spectrum scans of Tel33 after incubation in the absence of KCl (0 h, no salt) or with 50 mM KCl for 0.5, 8, or 24 h.
(EPS)

S2 Fig 3’ tail requirements are determined for G4R1 resolution of tetramolecular G4-DNA. (A) Representative resolvase assay in which TAMRA-end-labeled tetramolecular G4-DNA is unwound into monomeric oligomers and monitored by nondenaturing gel electrophoresis as previously described (38, 39, 42). (B) Tetramolecular G4- DNA sequences used in S2A Fig.
(EPS)
